# Carbohydrate-Energy Replacement Following High-Intensity Interval Exercise Blunts Next-Day Glycemic Control in Untrained Women

**DOI:** 10.3389/fnut.2022.868511

**Published:** 2022-03-22

**Authors:** Stephanie Estafanos, Beata Friesen, Alexa Govette, Jenna B. Gillen

**Affiliations:** Faculty of Kinesiology and Physical Education, University of Toronto, Toronto, ON, Canada

**Keywords:** low-volume interval exercise, energy deficit, carbohydrate deficit, blood glucose, continuous glucose monitor

## Abstract

**Background:**

Improved glycemic control has been reported for ∼24 h following low-volume high-intensity interval exercise (HIIE), but it is unclear if this is a direct effect of exercise or an indirect effect of the exercise-induced energy deficit. The purpose of this study was to investigate the effect of carbohydrate-energy replacement after low-volume HIIE on 24 h glycemic control in women.

**Methods:**

Seven untrained women (age: 22 ± 2 yr; BMI: 22 ± 3 kg/m^2^; VO_2_peak: 33 ± 7 ml/kg/min) completed three 2-day trials in the mid-follicular phase of the menstrual cycle. Continuous glucose monitoring was used to measure blood glucose concentrations during, and for 24 h following three conditions: (1) HIIE followed by a high-carbohydrate energy replacement drink (EX-HC); (2) HIIE followed by a non-caloric taste-matched placebo drink (EX-NC); and (3) seated control with no drink (CTL). HIIE involved an evening session (1,700 h) of 10 × 1-min cycling efforts at ∼90% maximal heart rate with 1 min recovery. Diet was standardized and identical across all three 2-day trials, apart from the post-exercise carbohydrate drink in EX-HC, which was designed to replenish the exercise-induced energy expenditure. Postprandial glycemic responses to the following days breakfast, snack, lunch, and dinner, as well as 24 h indices of glycemic control, were analyzed.

**Results:**

The day after HIIE, postprandial glycemia following breakfast and snack were reduced in EX-NC compared to EX-HC, as reflected by lower 3 h glucose mean (breakfast: 5.5 ± 0.5 vs. 6.7 ± 1, *p* = 0.01, Cohen’s *d* = 1.4; snack: 4.9 ± 0.3 vs. 5.7 ± 0.8 mmol/L, *p* = 0.02, *d* = 1.4) and/or area under the curve (AUC) (breakfast: 994 ± 86 vs. 1,208 ± 190 mmol/L x 3 h, *p* = 0.01, *d* = 1.5). Postprandial glycemic responses following lunch and dinner were not different across conditions (*p* > 0.05). The 24 h glucose mean (EX-NC: 5.2 ± 0.3 vs. EX-HC: 5.7 ± 0.7 mmol/L; *p* = 0.02, *d* = 1.1) and AUC (EX-NC: 7,448 ± 425 vs. EX-HC: 8,246 ± 957 mmol/L × 24 h; *p* = 0.02, *d* = 1.1) were reduced in EX-NC compared to EX-HC.

**Conclusion:**

Post-exercise carbohydrate-energy replacement attenuates glycemic control the day following a single session of low-volume HIIE in women.

## Introduction

Low-volume high-intensity interval exercise (HIIE), characterized by brief, repeated bouts of intense exercise separated by periods of rest or low-intensity recovery, improves glycemic control despite a low exercise volume (1, 2). For example, a single session of low-volume HIIE involving 8–10 × 1-min cycling intervals at ∼90% maximal heart rate (HRmax) has been demonstrated to reduce mean blood glucose concentrations and postprandial glycemic excursions for up to 24 h following exercise in adults with (3) and without (4, 5) type 2 diabetes. While acute improvements in glycemic control following low-volume HIIE have been established, there is limited information on whether post-exercise nutrition strategies can influence the magnitude and/or duration of the response.

In studies demonstrating improved glycemic control for up to 24 h following a single session of low-volume HIIE (3–5), the meals consumed on both exercise and non-exercise control days were the same. By design, this results in participants being in an exercise-induced energy deficit following HIIE, which may not reflect the effects of HIIE under conditions of energy balance, such as when a post-exercise snack is consumed during recovery. Indeed, compared to maintaining the energy deficit following a single session of moderate-intensity continuous exercise (MICE) (∼45–90 min at 60–70% of peak oxygen uptake; VO_2_peak), immediately refeeding the calories expended during exercise has been shown to attenuate insulin sensitivity (6, 7) and glycemic control (8) when measured ∼12–15 h post-exercise. Blunted exercise-induced improvements in insulin sensitivity and glycemic control under conditions of energy balance may be specific to moderate-intensity exercise however, as high-intensity (84% VO_2_peak) but not moderate-intensity (50% VO_2_peak) exercise with energy replacement was shown to improve insulin sensitivity 22 h post-exercise (9). These findings suggest that high-intensity exercise may improve insulin sensitivity independent of energy balance. However, the authors acknowledge that despite participants being in energy balance prior to assessment of insulin sensitivity, they may have been in a carbohydrate deficit, owing to the glycogen-dependent nature of high-intensity exercise (9). Indeed, the carbohydrate- rather than energy-deficit has been reported as the primary mediator of exercise-induced improvements in insulin sensitivity, owing to the maintenance of low muscle glycogen concentrations (6). Thus, it remains to be determined how high-intensity exercise under conditions of carbohydrate balance influence glycemic control.

Our understanding of the importance of the carbohydrate deficit following MICE is based on studies conducted in men (6, 10, 11) or mixed cohorts of men and women (7, 8, 12, 13). However, sex-based differences in metabolism can influence exercise and nutritional strategies for improved health and performance (14). Given that women have been reported to use less muscle glycogen during exercise (15) and experience blunted exercise-induced improvements in insulin sensitivity and glycemic control (16) compared to men, additional research is needed to inform exercise and nutrition recommendations for improved metabolic health in women. The purpose of the present study was to investigate the effect of carbohydrate-energy replacement after a single session of low-volume HIIE on postprandial and 24 h glycemic control in women. We hypothesized that when compared to maintaining the exercise-induced energy deficit following HIIE, post-exercise carbohydrate-energy replenishment would attenuate next-day postprandial and 24 h glycemic control.

## Materials and Methods

### Participants and Sample Size Estimation

Healthy women between the ages of 18–35 years were recruited using poster and web-based advertisements in the Greater Toronto Area (Table 1). Participants were deemed untrained based on a VO_2_peak classified as “good” or lower according to age- and sex-specific norms (18–29 year: ≤ 43.9 ml/kg/min) (17), performing < 150 min moderate-intensity physical activity per week, and not training for any specific sport. Participants were eumenorrheic, with a self-reported menstrual cycle length between 28 and 35 days, and not currently taking, or have taken, oral contraceptives in the last 6 months. Exclusion criteria included performing HIIE > 1 day/week, tobacco and/or illicit drug use, a diagnosed medical condition under the care of a physician, and/or currently taking medications that may affect substrate metabolism (e.g., corticosteroids, nSAIDs). Eligible participants were informed of the study purpose, experimental procedures, and potential risks before obtaining written informed consent. The study protocol was approved by the University of Toronto Health Sciences Research Ethics Board (protocol No. 00037680).

**TABLE 1:** Participant characteristics.

Variable	Value
Participants (*n*)	7
Age (y)	22 ± 2
Height (cm)	162 ± 6
Body mass (kg)	57 ± 7
BMI (kg⋅m^2–1^)	22 ± 3
Body fat (%)	27 ± 5
REE (kcal⋅d)	1,410 ± 79
VO_2_peak (ml⋅kg^–1^⋅min^–1^)	33 ± 7
Maximum power output (W)	197 ± 33
Workload for HIIE (W)	160 ± 28

*Values are expressed as means ± SD. BMI, body mass index; REE, resting energy expenditure; VO_2_peak, peak oxygen uptake.*

Sample size estimates were determined in G*Power (Version 3.1, Department of Psychology, Germany) using a previously reported effect size (Cohen’s *d* = 1.38) for the difference in next-day postprandial glucose area under the curve (AUC; primary outcome) following MICE with or without post-exercise carbohydrate replenishment (7). To detect a significant difference (*p* ≤ 0.05) between conditions with 80% power, it was calculated that *n* = 9 was required. Data collection took place between October 2019 and March 2020. As a result of the University’s mandatory laboratory closure in March 2020 due to the COVID-19 pandemic, only *n* = 7 participants completed the study and are included in the present manuscript.

### Baseline Testing

At least 1 week prior to experimental trials, participants reported to the laboratory following a 10-h overnight fast. Body composition was measured using air displacement plethysmography (BodPod; Cosmed USA Inc., Concord, CA) for determination of body mass and% body fat. Participants were then offered a 10% carbohydrate solution (Gatorade; PepsiCo, United States) prior to performing a VO_2_peak test on a cycle ergometer (Velotron DynaFit Pro; Racer-Mate, Seattle, WA). Expired gases were collected using a metabolic cart with an on-line gas collection system (TrueOne^®^ 2400; Parvo Medics, Salt Lake City, United States), and heart rate (HR) was measured continuously using a chest-worn strap (Polar A3; Polar, Lake Success, NY). Following a 5-min warm-up at 50 watts (W), the resistance was increased by 1 W every 2 s until volitional exhaustion. VO_2_peak and maximal workload (Wmax) were defined as the highest oxygen consumption and power output values achieved over a 30-s period, respectively. All participants achieved a respiratory exchange ratio above 1.1 by the end of the test. Following a brief period of recovery (∼10–15 min), participants completed a HIIE familiarization session involving 4 × 1-min intervals at ∼90% HRmax. Individual adjustments including seat height/angle, foot position, and handlebar height, were identified and replicated for subsequent trials.

### Experimental Trial Overview

**FIGURE 1:**
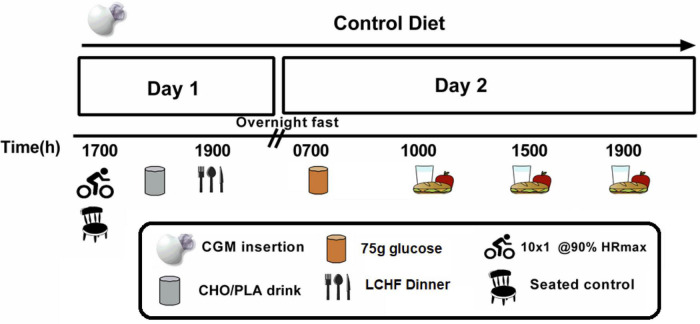
Schematic representation of metabolic trials. A continuous glucose monitor (CGM) was worn for three 2-day trials under standardized dietary intake. At 17:00 h on Day 1, participants either sat in the laboratory (CTL) or performed low-volume HIIE (10 × 1-min cycling intervals at ∼90% HRmax). Following HIIE, participants consumed either a carbohydrate (CHO) drink to replenish the exercise-induced energy deficit (EX-HC) or a non-caloric placebo (PLA) drink to remain in an exercise-induced energy deficit (EX-NC). All participants consumed a low-carbohydrate high-fat (LCHF) dinner that evening. Glycemic responses to the following day’s meals, and over a 24 h period (from 00:00 to 23:59 h), were determined *via* CGM.

A schematic representation of experimental trials is presented in Figure 1. Using a repeated-measures crossover design, participants completed three 2-day experimental conditions: (1) HIIE followed by a high-carbohydrate energy replacement drink (EX-HC); (2) HIIE followed by a non-caloric taste-matched placebo drink (EX-NC); and (3) seated control with no drink (CTL). All experimental conditions were conducted in the early- to mid-follicular phase of the menstrual cycle (days 3–9), in accordance with best practice guidelines for including women in physiological research (18). To ensure sufficient washout between exercise trials (EX-HC and EX-NC), participants conducted these trials in two separate menstrual cycles, ∼4 weeks apart. The CTL trial was conducted in advance of one of these trials (with 1 day of washout in between), which allowed for testing in the early- to mid-follicular phase, but mitigated variability that could be introduced by extending testing over 3 menstrual cycles, or ∼8 weeks. Both the order of the exercise trials (EX-NC and EX-HC), and whether the CTL trial was performed in advance of the first or second exercise trial, was randomized between participants using an online platform (Research Randomizer).

Participants were instructed to refrain from any structured physical activity for 2 days before, and throughout, all experimental trials. One day before experimental trials, participants reported to the laboratory at ∼16:00 h for insertion of a continuous glucose monitor (CGM) (iPro2; Medtronic, Northridge, CA) into the subcutaneous tissue of the abdomen. Participants were also given a pre-packaged standardized diet to consume over the subsequent 2-day metabolic trial. On Day 1 of the metabolic trial, participants consumed a standardized mixed-macronutrient breakfast (09:00 h), lunch (12:00 h), and snack (15:00 h) under “free-living” conditions (i.e., outside of the laboratory). Participants reported to the laboratory at 17:00 h and either performed a supervised session of HIIE (10 × 1-min cycling intervals at ∼90% HRmax; EX-HC and EX-NC) or remained seated at a desk (CTL). After exercise, EX-HC consumed a carbohydrate beverage equivalent to their exercise-induced energy expenditure, and EX-NC consumed a taste-matched non-caloric placebo beverage. Following, participants were seated in the laboratory before consuming a low-carbohydrate high-fat (LCHF) dinner meal at 19:00 h. Participants left the laboratory at 20:00 h and abstained from consuming any food or drink other than water for the remainder of the evening. On Day 2, participants reported to the laboratory at 07:00 h following a 10-h overnight fast. An indwelling catheter was inserted into a forearm vein, which was followed by the consumption of a 75 g glucose beverage (NERL™, Thermo Fisher, CAN) as breakfast. Over the subsequent 3 h, repeated blood samples were obtained every 30 min while participants were seated at a desk. At 10:00h, participants consumed a mixed-macronutrient snack prior to leaving the laboratory, which was followed by consumption of a standardized lunch (13:00 h) and dinner (18:00 h) under “free-living” conditions. Postprandial responses to all meals, as well as over 24 h, were measured *via* CGM. Owing to difficulties in obtaining blood samples from 5/7 female participants due to loss of the intravenous catheter in at least one of their trials, and hemolyzed blood samples in 4/7 participants, blood sample analyses in response to breakfast was not included in the present manuscript.

### Experimental Diet Composition

Daily energy intake was determined according to the calculation of resting energy expenditure (REE) using the Harris-Benedict equation (19) multiplied by a physical activity correction of 1.4 (20). Standardized meals were provided for participants to consume at specific times during metabolic trials. The diet across all metabolic trials was identical within each participant, apart from the post-exercise drink in exercise trials (EX-HC/EX-NC). On Day 1, ∼30% of daily energy intake was provided at breakfast, lunch, and dinner, with the remaining ∼10% of daily energy provided as a mid-afternoon snack (Table 2). Breakfast (09:00 h), lunch (12:00 h), and snack (15:00 h) were consumed under “free-living” conditions and had a macronutrient composition of ∼55% carbohydrate, ∼30% fat and ∼15% protein. All meals provided contained pre-packaged (e.g., yogurt, granola bar, juice box) and/or easy to prepare (e.g., turkey and cheese sandwich, carrot sticks) items from the local grocery store. On the evening of Day 1 (19:00 h), a LCHF dinner was consumed in the laboratory following exercise or sitting, and consisted of a salmon filet, guacamole, macadamia nuts, and cesar salad containing romaine lettuce, bacon bits, and garlic croutons (∼5/80/15% carbohydrate/fat/protein). On the morning of Day 2, participants consumed a 75 g glucose beverage in the laboratory in replace of breakfast (07:00 h), equating to ∼15% of daily energy needs. The remainder of Day 2 meals were pre-packaged mixed-macronutrient meals consumed under free-living conditions and provided as snack (∼15% of daily energy intake; 10:00 h), lunch (∼35%; 13:00 h) and dinner (∼35%; 18:00 h). For all meals consumed under “free-living” conditions, compliance was assessed by asking participants to report the timing of each meal and checkmark each food item as it was consumed on the personalized diet sheets provided.

**TABLE 2:** Nutritional composition of standardized meals.

Meal	Energy (kcal)	CHO (g)	Fat (g)	Protein (g)
**Day 1 meals**				
Breakfast (∼30% EI)	593 ± 19	82 ± 3	20 ± 0	21 ± 1
Lunch (∼30% EI)	598 ± 29	81 ± 5	21 ± 1	22 ± 2
Snack (∼10% EI)	198 ± 18	28 ± 2	6 ± 1	8 ± 1
LCHF Dinner (∼30% EI)	609 ± 26	8 ± 0	53 ± 3	24 ± 0
**Total daily intake**	1,997 ± 84	199 ± 9	100 ± 4	75 ± 3
**Day 2 meals**				
Breakfast (∼15% EI)	300	75	0	0
Snack (∼15% EI)	300 ± 35	41 ± 5	10 ± 1	12 ± 1
Lunch (∼35% EI)	675 ± 32	94 ± 5	22 ± 1	25 ± 2
Dinner (∼35% EI)	694 ± 44	94 ± 6	23 ± 2	28 ± 1
**Total daily intake**	1,969 ± 111	304 ± 15	55 ± 4	65 ± 4

*Values are expressed as means ± SD.*

*CHO, carbohydrate; EI, energy intake; LCHF, low-carbohydrate high-fat.*

### Exercise Protocol

Following a 5 min warm-up at 50 W, participants completed 10 × 1-min cycling intervals at ∼90% HRmax (∼80% Wmax) with 1-min recovery in between (light cycling at 50 W) on a cycle ergometer (Velotron DynaFit Pro; Racer-Mate, Seattle, WA), followed by a 5-min cool down at 50 W, as previously described (21). Oxygen consumption (VO_2_) was measured *via* indirect calorimetry (TrueOne^®^ 2400; Parvo Medics, Salt Lake City, United States), and HR was monitored continuously using a chest-worn strap (Polar A3, Lake Success, NY). Rating of perceived exertion (RPE) was recorded after each interval using the Borg 0–10 category/ratio scale (22). Strong verbal encouragement was provided during all trials.

### Post-exercise Nutrition

In EX-HC, participants ingested a 15% carbohydrate solution containing 50% Polycal^®^ (Nutricia, United Kingdom) and 50% Gatorade power (PepsiCo, United States) immediately after HIIE (within 9 ± 4 min). This beverage was prepared by the lead investigator and was designed to precisely replenish the exercise-induced energy expenditure measured *via* indirect calorimetry (43.4 ± 9.8 g carbohydrate; 173 ± 39 kcal). The exercise-induced energy expenditure was calculated following HIIE by subtracting resting energy expenditure over 29 min (assuming 3.5 mL O_2_/kg/min) from total energy expenditure measured during exercise (assuming 5 kcal expended per 1 L O_2_ consumed). During EX-NC, participants ingested an artificially sweetened non-caloric placebo beverage (MiO; Kraft Heinz, Canada) to remain in an energy and carbohydrate deficit. No beverage was provided in CTL.

### Continuous Glucose Monitor

CGM was used for measurement of blood glucose concentration throughout each 2-day trial. CGM captures glycemic responses to real meals under “free-living” conditions, which provides information on glycemic control outside of a laboratory setting (23). The single-use sensor, which records interstitial glucose concentrations every 5 min, was connected to a monitor placed on the abdomen (iPro2; Medtronic, Northridge, CA). The same iPro2 monitor was used across repeat trials for each participant and manufacturer’s recommendations were followed while in use. This included having participants measure their capillary blood glucose concentration (OneTouch UltraMini, Lifescan, Milpitas, CA) four times each day when blood glucose was expected to be stable (upon awakening, before lunch, before dinner, and before bed) for calibration purposes, consistent with our previous work (3, 8). Research in adults without diabetes has found CGM-derived postprandial glycemic responses to be reproducible (24) and comparable to both intravenous- and capillary-derived glucose measurements (25).

### Analytical Procedures

Interstitial glucose data stored within CGM was uploaded to an online program (CareLink Pro Management Software; Medtronic, Northridge, CA) which, when combined with the finger stick capillary glucose calibration points, generated blood glucose concentrations every 5 min for the duration in which the CGM was worn. Blood glucose concentrations were exported to Microsoft Excel for initial inspection, and subsequently transferred to GraphPad Prism (GraphPad Software V8; San Diego, CA, United States) for graphing and analyses. The day following exercise or control (Day 2; referred to as “next day” throughout), postprandial glycemic responses were assessed by calculating the 3 h glucose average, peak, and AUC using the trapezoidal rule, as we have described previously (3, 26). 12 h overnight glycemia (defined as the 12 h period prior to next-day breakfast) and 24 h glucose mean, AUC and glycemic variability (defined as the 24 h period from 00:00 to 23:59 h on Day 2) were also calculated. Indices of glycemic variability included: (1) mean amplitude of glycemic excursions (MAGE); (2) continuous overall net glycemic action (CONGA); (3) standard deviation (SD); and (4) % coefficient of variation (CV). SD and CV were calculated in excel as previously described (27, 28) and MAGE and CONGA were assessed using an online platform (EasyGV)^1^ (29).

In addition to the primary next-day outcomes of interest, select CGM-derived blood glucose responses on Day 1 were analyzed, including the change in glucose concentration from pre- to post-exercise, and the 1 h mean glucose concentration following the post-exercise drinks.

### Statistical Analysis

Statistical analyses were performed using GraphPad Prism (Version 8; GraphPad Software, San Diego, CA, United States). Normality of the data was confirmed using the Shapiro-Wilk test. Missing CGM data points (1 participant in EX-HC owing to sensor malfunction midway through the trial) were replaced with the series mean for that timepoint. A one-way repeated measures analysis of variance (ANOVA) was used to determine main effects of condition (EX-HC, EX-NC, CTL) for next-day indices of glycemic control and day tested of menses. If a significant ANOVA was obtained, a Fisher’s Least Significant Differences (LSD) *post-hoc* test was performed to identify differences between conditions. Fisher’s LSD preserves the type I error rate relatively well when the number of treatment groups is equal to three (as in our study) (30), and thus was selected *a priori*, consistent with others (8, 12). Paired samples *t*-tests were used to analyze differences between exercise trials (EX-HC and EX-NC) for exercise-related outcomes (HR and VO_2_) and post-exercise glycemia in response to the test drink. The change in mean glucose concentration from pre- to post-exercise was analyzed by a two-way repeated measures ANOVA with time (pre vs. post) x condition (EX-HC vs. EX-NC). The level of significance for all analyses was set at *p* ≤ 0.05 and data for all outcomes reflect *n* = 7. Data are presented as means ± *SD*. Cohen’s *d* effect sizes were calculated for pairwise differences in next-day glycemia with 0.2, 0.5 and 0.8 considered the thresholds for small, medium, and large effect sizes, respectively.

## Results

### Participant Characteristics and Responses to High-Intensity Interval Exercise and Post-exercise Nutrition

Participant characteristics are summarized in Table 1. Participants were tested on day 6 ± 2 of their menstrual cycle (mean cycle length: 29 ± 2 days, range: 25–31 days), with no difference between trials (CTL: 6 ± 1, EX-HC: 6 ± 2, EX-NC: 7 ± 2; *p* = 0.61). All participants completed the HIIE protocol in both trials with 100% compliance. Mean RPE (EX-HC: 7.7 ± 0.3, EX-NC: 7.8 ± 0.7; *p* = 0.53) and%VO_2_peak (EX-HC: 89.1 ± 4.5, EX-NC: 91.1 ± 4.7%; *p* = 0.22) were similar between trials, but a 1% difference in % HRmax reached statistical significance (EX-HC: 90.1 ± 3.2, EX-NC: 89.1 ± 2.6%; *p* = 0.03). Exercise-induced VO_2_ was similar in EX-HC (34.7 ± 7.9) and EX-NC (36.5 ± 5.9 L; *p* = 0.19), resulting in comparable estimates of exercise-induced energy expenditure between conditions (EX-HC: 173.3 ± 39.4, EX-NC: 182.5 ± 29.7 kcal; *p* = 0.19). CGM-derived blood glucose concentration increased from pre- (5.0 ± 0.6) to post-exercise (5.5 ± 0.9 mmol/L) with no difference between trials (main effect of time, *p* = 0.046). The 1 h glycemic excursion following the post-exercise drink was greater in EX-HC (mean: 6.6 ± 1; AUC: 397 ± 60) compared to EX-NC (mean: 5.3 ± 0.6 mmol/L, *p* = 0.04; AUC 315 ± 38 mmol/L x 1 h, *p* = 0.04).

### Glycemic Control

#### Next-Day Postprandial Glycemia

**FIGURE 2:**
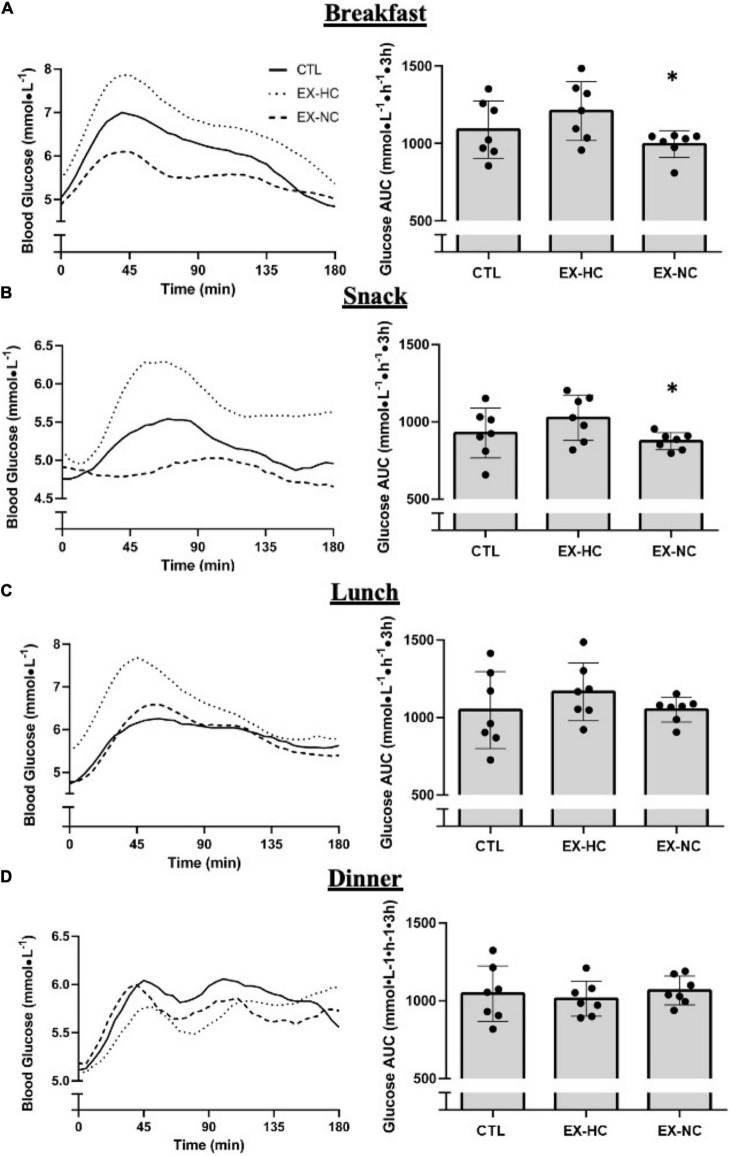
Glycemic responses 3 h following breakfast **(A)**, snack **(B)**, lunch **(C)** and dinner **(D)**, measured the day following seated control (CTL) or a single session of HIIE with carbohydrate-energy replacement (EX-HC) or deficit (EX-NC). *Significant difference from EX-HC (*p* < 0.05); Values are expressed as means ± *SD*.

Following breakfast, 3 h postprandial glycemia was lower in EX-NC compared to EX-HC (glucose mean: *p* = 0.01, *d* = 1.4; glucose AUC: *p* = 0.01, *d* = 1.5; Table 3 and Figure 2). In response to snack, the 3 h mean blood glucose concentration was also lower in EX-NC relative to EX-HC (*p* = 0.02, *d* = 1.4; Table 3). A main effect of condition for 3 h AUC following snack approached significance (*p* = 0.058), which, if followed up with pairwise comparisons, revealed a reduction in EX-NC relative to EX-HC (*p* = 0.02, *d* = 0.64; Figure 2). Glycemic responses to breakfast and snack were not different between either exercise condition relative to CTL (*p* > 0.05); however, medium effect sizes favored reduced postprandial glucose mean (*p* = 0.24, *d* = 0.64) and AUC (*p* = 0.23, *d* = 0.65) at breakfast in EX-NC relative to CTL (Table 3 and Figure 2). Peak glucose concentration did not differ among conditions at breakfast or snack (Table 3). Postprandial glycemic responses to lunch and dinner meals were not different among conditions (*p* > 0.05; Table 3 and Figure 2).

**TABLE 3:** Analysis of CGM-derived glycemic outcomes.

	CTL	EX-HC	EX-NC
**Breakfast**			
Mean glucose (mmol⋅L^–1^)	6.0 ± 1.0	6.7 ± 1.0	5.5 ± 0.5*
Peak glucose (mmol⋅L^–1^)	7.7 ± 1.6	8.0 ± 1.6	7.0 ± 0.9
**Snack**			
Mean glucose (mmol⋅L^–1^)	5.1 ± 0.9	5.7 ± 0.8	4.9 ± 0.3
Peak glucose (mmol⋅L^–1^)	5.9 ± 1.3	6.5 ± 1.1	5.5 ± 0.4
**Lunch**			
Mean glucose (mmol⋅L^–1^)	5.8 ± 1.4	6.5 ± 1.0	5.8 ± 0.4
Peak glucose (mmol⋅L^–1^)	6.8 ± 1.7	7.9 ± 1.6	7.2 ± 0.6
**Dinner**			
Mean glucose (mmol⋅L^–1^)	5.8 ± 1.0	5.7 ± 0.6	5.7 ± 0.5
Peak glucose (mmol⋅L^–1^)	6.7 ± 1.4	6.4 ± 0.8	6.4 ± 0.5
**Overnight**			
Mean glucose (mmol L^–1^)	5.0 ± 1.0	5.3 ± 1.0	4.9 ± 1.0
Glucose AUC (mmol⋅h^–1^⋅12h)	3,627 ± 412	3,773 ± 316	3,507 ± 272
**24 h Glycemia**			
Mean glucose (mmol⋅L^–1^)	5.4 ± 0.7	5.7 ± 0.7	5.2 ± 0.3*
CONGA (mmol⋅L^–1^)	4.8 ± 0.7	5.2 ± 0.5	4.7 ± 0.4*
MAGE (mmol⋅L^–1^)	2.1 ± 1.1	2.4 ± 0.9	2.0 ± 0.5
SD (arbitrary units)	0.8 ± 3.7	0.8 ± 0.3	0.8 ± 0.2
CV (%)	14.3 ± 6.4	13.2 ± 3.6	15.5 ± 4.1

*Postprandial data represents the 3 h period after each meal. Overnight data was analyzed from ∼19:00 to 07:00 h and 24 h data was analyzed from 00:00 to 23:59 h.*

*Significant difference from EX-HC (p < 0.05). Values are expressed as means ± SD.

*CONGA, continuous overall net glycemic action; MAGE, mean amplitude of glycemic excursions; SD, standard deviation; CV, % coefficient of variation.*

#### Next-Day 24 h Glycemia

**FIGURE 3:**
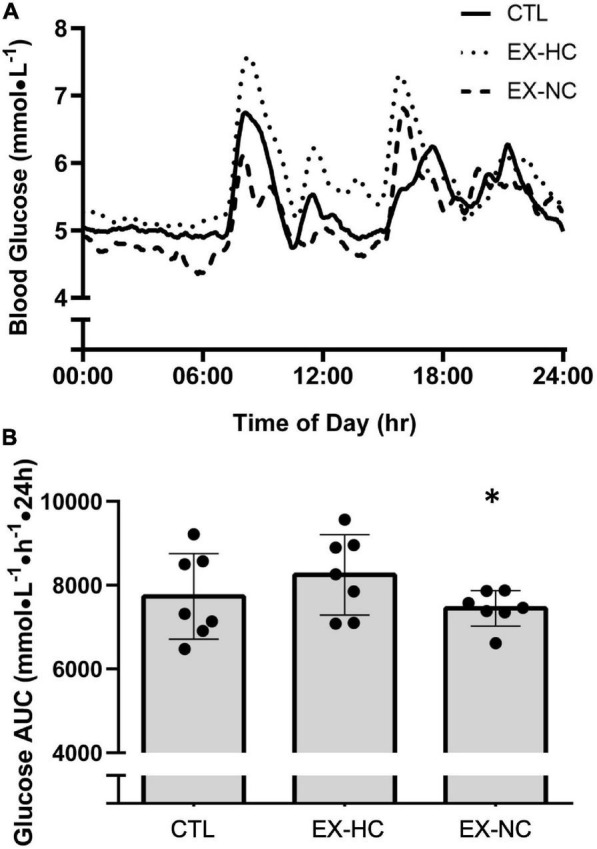
24 h blood glucose concentrations from 00:00 **to** 23:59 h, measured the day following seated control (CTL) or a single session of HIIE with carbohydrate-energy replacement (EX-HC) or deficit (EX-NC) **(A)**. Glucose AUC over the 24 h measurement period during experimental conditions **(B)**. *Significant difference from EX-HC (*p* < 0.05); Values are expressed as means ± *SD*.

Next-day 24-h blood glucose concentrations are presented in Figure 3. Compared to EX-HC, EX-NC had significantly lower 24 h mean blood glucose concentration (*p* = 0.02, *d* = 1.1) and 24 h glucose AUC (*p* = 0.02, *d* = 1.1; Table 3 and Figure 3). The 24 h mean blood glucose concentration and glucose AUC were not different between CTL and EX-NC (mean: *p* = 0.34, *d* = 0.36; AUC: *p* = 0.34, *d* = 0.36), or CTL and EX-HC (mean: *p* = 0.11, *d* = 0.52; AUC: *p* = 0.11, *d* = 0.52; Table 3 and Figure 3). Indices of glycemic variability (MAGE, SD, % CV) were not different across trials except for CONGA, which was lower in EX-NC compared to EX-HC (*p* = 0.02, *d* = 1.2; Table 3).

#### Overnight Glycemia

There was no statistical difference in overnight glycemia between conditions for 12 h glucose mean (*p* = 0.19) and AUC (*p* = 0.19); however, large effect sizes favored reduced overnight glucose mean (*d* = 0.93) and AUC (*d* = 0.90) in EX-NC relative to EX-HC (Table 3).

## Discussion

Low-volume HIIE has been reported to reduce mean blood glucose concentrations and postprandial glycemic excursions for up to 24 h in adults with (3) and without (4, 5) type 2 diabetes. In these studies, diet was carefully matched between the exercise and control trials, but the meals and/or snacks consumed after exercise did not compensate for the exercise-induced energy expenditure. By design, this results in an energy deficit during the HIIE condition only, making it difficult to discern the importance of the energy deficit vs. exercise *per se*, on the post-exercise improvement in glycemic control. In the present study, we sought to determine the importance of the HIIE-induced energy deficit by implementing two exercise trials that differed in whether the exercise-induced energy expenditure was replenished with a post-exercise carbohydrate drink. Consistent with our hypothesis, we observed that refeeding the exercise-induced energy expenditure with carbohydrate post-exercise blunted 24 h glycemia and postprandial glycemic excursions to breakfast and snack the next day, relative to when the exercise-induced energy deficit was maintained. These results expand our understanding of the influence of exercise on metabolic health as they suggest that glycemic control the day after low-volume HIIE is influenced by post-exercise nutritional intake.

Our findings are consistent with previous research demonstrating that improvements in insulin sensitivity and glycemic control following MICE are blunted when post-exercise meals contain carbohydrate (6–8, 12, 13). For example, compared to remaining in an energy deficit following 90 min of running at 70% VO_2_max, refeeding the exercise-induced energy expenditure with carbohydrate (∼221 g) attenuated oral glucose tolerance test-derived insulin sensitivity the next morning in healthy adults (11 men, 3 women) (7). In the present study, participants replenished the HIIE-induced energy deficit solely with carbohydrates (∼43 g) as high-intensity exercise relies almost exclusively on carbohydrates for energy provision (31). By virtue of our design, we cannot distinguish between the importance of the carbohydrate- vs. energy-deficit *per se*, similar to others (7, 8, 12). However, previous research has demonstrated that while carbohydrate intake blunts exercise-induced improvements in insulin sensitivity (6), post-exercise meals lacking carbohydrate, but containing fat and protein, do not blunt next-day insulin sensitivity following MICE (6, 10, 32). For example, despite creating an energy surplus *via* the provision of fat following 90 min of running at 65% VO_2_peak, next-day glucose tolerance and insulin sensitivity were not impaired (10, 32). Taken together, remaining in a negative carbohydrate balance, rather than a negative energy balance, appears critical for sustaining exercise-induced improvements in insulin sensitivity and glycemic control, most likely attributed to the maintenance of reduced muscle glycogen concentrations (11). While muscle biopsies were not obtained in the present study, the HIIE protocol implemented in this study has been shown to lower muscle glycogen stores by ∼25% in healthy adults (33). Given that carbohydrate intake was limited to ∼8 g at dinner in the energy deficit (EX-NC) trial, this would have been insufficient to replenish muscle and/or liver glycogen concentrations following HIIE.

The use of CGM in the present study allowed for novel characterization of the time-course of glycemic improvements the day following low-volume HIIE with or without carbohydrate replenishment. The majority of previous research evaluating the impact of the carbohydrate-energy deficit have used oral- (7, 10, 12) or intravenous- (6, 32) derived measurements of insulin sensitivity the following morning which, while valid laboratory-based measurement tools, provide only a “snapshot” into the effects of exercise on glucose tolerance. Using CGM, we demonstrated an improvement in next day 24 h glycemia in EX-NC relative to EX-HC, which was measured outside of the laboratory under “free-living” conditions. The glycemic improvement in EX-NC was seemingly reduced throughout the 24 h measurement period, however, as postprandial glycemia was improved following breakfast and snack, but not lunch and dinner. These findings are consistent with Schleh et al. (8) who observed improved postprandial glycemia at breakfast, but not lunch and dinner, the day following cycling that expended 350 kcal (8). By way of an explanation, it has been demonstrated that exercise-induced improvements in insulin sensitivity may be apparent until muscle glycogen is restored to baseline (34, 35). We anticipate that the carbohydrate content of breakfast (75 g) and snack (∼40 g) in our study were sufficient to restore muscle glycogen in EX-NC, further supporting the sensitivity of acute exercise-induced improvements in glycemia to carbohydrate intake.

From a practical perspective, our findings support the importance of considering the effects of meals consumed after exercise when exercising for improved glycemic control. Previous research suggests that many adults believe that high-carbohydrate foods should be consumed immediately post-exercise in an effort to replenish carbohydrate stores (36). This notion may stem from the often-publicized benefits of post-exercise carbohydrate consumption for muscle glycogen restoration, which is important for improved recovery and sport performance. However, many novice and recreational exercisers engage in physical activity to improve their health, and optimal post-exercise nutrition for these goals may be different from that which is recommended to enhance sport performance. Indeed, our findings suggest that restricting, rather than augmenting, the carbohydrate content of the meals and/or beverages consumed post-exercise results in better next-day glycemic control. While our proof-of-concept study also had participants maintain an energy deficit post-exercise, previous research suggests that this is not required for improvements in insulin sensitivity if the carbohydrate deficit is maintained (6, 9). In this regard, we (37, 38) and others (39, 40) have suggested that consuming low-carbohydrate and/or low-glycemic index meals post-exercise may optimize next-day glycemic control. Additional research is needed to determine how specific post-exercise foods high in fat and/or protein, but low in carbohydrate, influence glycemic control the day following low-volume HIIE.

Despite differences in next-day glycemic control between the two exercise conditions, we did not detect a significant improvement in next-day glycemic control in either exercise trial compared to the non-exercise control trial. This was unexpected as previous studies have reported acute exercise-induced improvements in glycemic control (3–5) and insulin sensitivity (41) following low-volume HIIE when compared to a non-exercise control. The discrepancy between our study and others may be due to our participants having a lower BMI (∼22 kg/m^2^) and higher fitness (∼33 ml/kg/min), which may blunt the acute effects of exercise on glycemic control (42, 43). Sex-based differences may also be involved as women have been reported to have blunted acute (44) and chronic (16) exercise-induced improvements in glycemic control relative to men. It is also possible that the low-carbohydrate evening meal created a favorable environment for next-day glycemia in the control condition, as postprandial metabolism at rest can be influenced by prior evening’s meal (45). Notwithstanding these possibilities that may explain the lack of statistical difference between the control and exercise trials, we did observe medium effect sizes for reduced postprandial glycemia in response to breakfast (mean: *d* = 0.64; AUC: *d* = 0.65) in EX-NC relative to CTL. Thus, it is also conceivable that our small sample size precluded our ability to detect a statistically significant effect of the exercise-induced carbohydrate-energy deficit relative to control, reflecting a potential type II error. Additional research is warranted that evaluates the acute effects of low-volume HIIE on peripheral insulin sensitivity and glycemic control in larger cohorts of women across the lifespan, including studies which include direct comparisons to men.

Our study design has a number of strengths, including the careful control of dietary intake which allowed us to demonstrate for the first time that the meals consumed after low-volume HIIE influence next-day glycemic control. To the best of our knowledge, we are also the first to demonstrate the importance of the carbohydrate-energy deficit on glycemic control in a cohort of women. It is well recognized that women are understudied in exercise and nutrition research relative to men (46), which has resulted in a lack of post-exercise nutritional recommendations specific to women. Our findings therefore advance the field in this regard and demonstrate the importance of considering the influence of post-exercise nutrition in future studies investigating acute and chronic changes to insulin sensitivity. It is possible that differences in post-exercise carbohydrate intake following acute exercise sessions partially explain the blunted training-induced improvements in glycemic control (16) and insulin sensitivity (47) previously observed in women relative to men. Additional research is needed to test this hypothesis, as is research exploring exercise-nutrient interactions in women with impaired glycemic control (e.g., obese and/or type 2 diabetes) as our results are specific to healthy individuals.

The present study involved a small sample size (*n* = 7), but one that is in line with other research (*n* = 7–9) investigating how macronutrient intake following MICE influences next-day insulin sensitivity (6, 10, 12). Nonetheless, our limited sample size may have predisposed our study to type II errors in some pairwise comparisons with CTL, as demonstrated by medium effect sizes for statistically non-significant differences between EX-NC and CTL (e.g., 3 h breakfast mean and AUC, *d* > 0.6). However, the primary aim of this study was to establish the extent to which replenishing the exercise-induced energy deficit with carbohydrate alters next-day glycemic control, which is demonstrated in the primary comparison between the two exercise trials. In addition, while our study demonstrates the sensitivity of exercise-induced improvements in glycemic control to post-exercise carbohydrate, we did not measure carbohydrate balance nor patterns of glycogen use and resynthesis across the liver and skeletal muscle between conditions. The amount of carbohydrate provided in the post-exercise drink in EX-HC was based on the exercise-induced energy expenditure measured with indirect calorimetry, consistent with others (7, 8, 12). However, this method does not capture the contribution of anaerobic energy metabolism, which has been estimated to represent approximately 30% of energy expenditure during low-volume HIIE (48). As muscle glycogen is the primary substrate for anaerobic metabolism during exercise, the carbohydrate content of our post-exercise drink based on the indirect calorimetry-derived aerobic energy deficit alone may have underestimated the amount needed to restore carbohydrate balance and muscle glycogen concentration. In this case, our findings would suggest that HIIE-induced improvements in glycemia are sensitive to even partial restoration of carbohydrate balance. Additional research that includes muscle biopsies is needed to clarify this and further characterize underlying mechanisms contributing to the observed differences in glycemia between conditions.

## Conclusion

In conclusion, our findings demonstrate that consuming a post-exercise carbohydrate beverage equivalent to the exercise-induced energy expenditure blunts 24 h mean and postprandial glycemia the day following low-volume HIIE in women, relative to when the exercise-induced carbohydrate deficit is maintained. Additional research is needed to explore whether alternative post-exercise snacks and/or meals that are low in carbohydrate can support the glycemic benefits of low-volume HIIE in women.

## Data Availability Statement

The raw data supporting the conclusions of this article will be made available by the authors, without undue reservation.

## Ethics Statement

The studies involving human participants were reviewed and approved by the University of Toronto Health Sciences Research Ethics Board. The patients/participants provided their written informed consent to participate in this study.

## Author Contributions

SE and JG conceived and designed the experimental protocol, analyzed all the data, performed the statistical analysis, and wrote the manuscript. SE, BF, AG, and JG involved in data collection procedures. All authors contributed to editing the manuscript.

## Conflict of Interest

The authors declare that the research was conducted in the absence of any commercial or financial relationships that could be construed as a potential conflict of interest.

## Publisher’s Note

All claims expressed in this article are solely those of the authors and do not necessarily represent those of their affiliated organizations, or those of the publisher, the editors and the reviewers. Any product that may be evaluated in this article, or claim that may be made by its manufacturer, is not guaranteed or endorsed by the publisher.

## Footnotes

1
http://www.phc.ox.ac.uk/research/technology-outputs/easygv
